# Tripterygium glycosides: recent advances in mechanisms, therapeutic applications, and safety optimization

**DOI:** 10.3389/fmed.2026.1728162

**Published:** 2026-02-02

**Authors:** Yujie Jin, Yongxin Cui, Zhanyan Zhang, Chenglin Huang, Ruoting Tong, Ye Ling, Qirui Pei, Yan Ma, Qixia Zhan, Xiaojian Leng, Junjun He, Lizhuo Wang, Jialin Gao

**Affiliations:** 1 Department of Endocrinology and Genetic Metabolism, The First Affiliated Hospital of Wannan Medical College (Yijishan Hospital of Wannan Medical College), Wuhu, Anhui, China; 2 Institute of Endocrine and Metabolic Diseases, The First Affiliated Hospital of Wannan Medical College (Yijishan Hospital of Wannan Medical College), Wuhu, Anhui, China; 3 Anhui Province Key Laboratory of Basic Research and Transformation of Age-Related Diseases, Wannan Medical College, Wuhu, Anhui, China; 4 Department of Biochemistry and Molecular Biology, Wannan Medical College, Wuhu, Anhui, China; 5 Guangde People's Hospital, Xuancheng, Anhui, China

**Keywords:** autoimmune diseases, diterpenoids, natural products, structural optimization, translational pharmacology, Tripterygium glycoside*s*, triptolide, triptonide

## Abstract

Tripterygium glycosides (TG), bioactive extracts derived from *Tripterygium wilfordii* Hook F., possess potent anti-inflammatory and immunomodulatory properties, making them promising therapeutic candidates for a range of autoimmune and inflammatory diseases. This review summarizes recent advances in the pharmacological mechanisms of TG, including their roles in cytokine suppression, autophagy modulation, anti-fibrotic remodeling, and oxidative stress regulation. Evidence from clinical trials and real-world studies supports the therapeutic potential of TG in conditions such as systemic lupus erythematosus, diabetic kidney disease, rheumatoid arthritis, and psoriasis. In addition, we highlight ongoing efforts to overcome TG's narrow therapeutic window through monomer isolation, structural optimization, prodrug strategies, and innovative delivery systems. Emerging derivatives—such as LLDT-8 (5R-5-hydroxytriptolide) and triptonide—exhibit reduced toxicity while retaining robust efficacy, providing new avenues for clinical translation. Furthermore, the integration of systems pharmacology, synthetic biology, and AI-assisted drug design is accelerating the development of next-generation TG-based therapeutics.

## 1 Introduction

*Tripterygium wilfordii* Hook F. (TwHF), also known as Thunder God Vine, is a long-standing herb in traditional Chinese medicine (TCM) and has been used for centuries to treat chronic inflammatory and autoimmune disorders. Among its various bioactive constituents, Tripterygium glycosides (TG)—a partially purified extract standardized from TwHF—have attracted increasing interest in modern pharmacological research. TG consists of multiple diterpenoids, triterpenoids, and alkaloids, and is widely used in clinical practice in China for conditions such as rheumatoid arthritis (RA), systemic lupus erythematosus (SLE), and chronic kidney diseases, particularly lupus nephritis and IgA nephropathy (1, 2).

Over the past two decades, substantial experimental and clinical evidence has demonstrated that TG exerts its therapeutic effects through multiple molecular and cellular mechanisms. These include inhibition of pro-inflammatory pathways (e.g., NF-κB, JAK/STAT, MAPK), modulation of immune cell activation (e.g., T cells, B cells, macrophages), and suppression of pro-fibrotic signaling cascades (e.g., TGF-β/Smad). Notably, TG also appears to modulate autophagy, attenuate oxidative stress, and induce apoptosis in aberrant immune or stromal cells. Together, these pleiotropic actions position TG as a unique immunomodulatory agent with broad translational potential (3). Recently, TG has regained significant research attention for its potential therapeutic applications in diabetic kidney disease (DKD), especially in cases where traditional therapies, such as renin–angiotensin–aldosterone system inhibitors and SGLT2 inhibitors, do not fully halt disease progression. Emerging evidence suggests that TG exerts anti-proteinuric, anti-inflammatory, and anti-fibrotic effects in DKD, thereby addressing important unmet clinical needs (4). Moreover, TG has demonstrated synergistic benefits when combined with modern therapies, providing a compelling rationale for its integration into combination treatment regimens.

Despite its clinical promise, the therapeutic window of TG remains narrow, and its dose-limiting toxicities—particularly hepatotoxicity, reproductive toxicity, and gastrointestinal intolerance—have hindered broader global adoption. To address these limitations, recent efforts have focused on optimizing TG formulations, developing targeted delivery systems, and identifying predictive biomarkers of toxicity to improve patient safety. Additionally, several TG-derived monomer compounds, such as triptolide and celastrol, are undergoing structural modification and preclinical evaluation to enhance efficacy while reducing toxicity (5). Given its complex pharmacology, evolving clinical utility, and the urgent need for safer and more effective immunoregulatory agents, TG represents a promising yet underutilized candidate for drug development. This review aims to summarize the latest advances in TG's mechanisms of action, therapeutic applications across multiple disease domains, safety challenges, and emerging innovation strategies that may help bridge the gap between its traditional use and modern global pharmaceutical development.

## 2 Pharmacological mechanisms of Tripterygium glycosides

**Figure 1:**
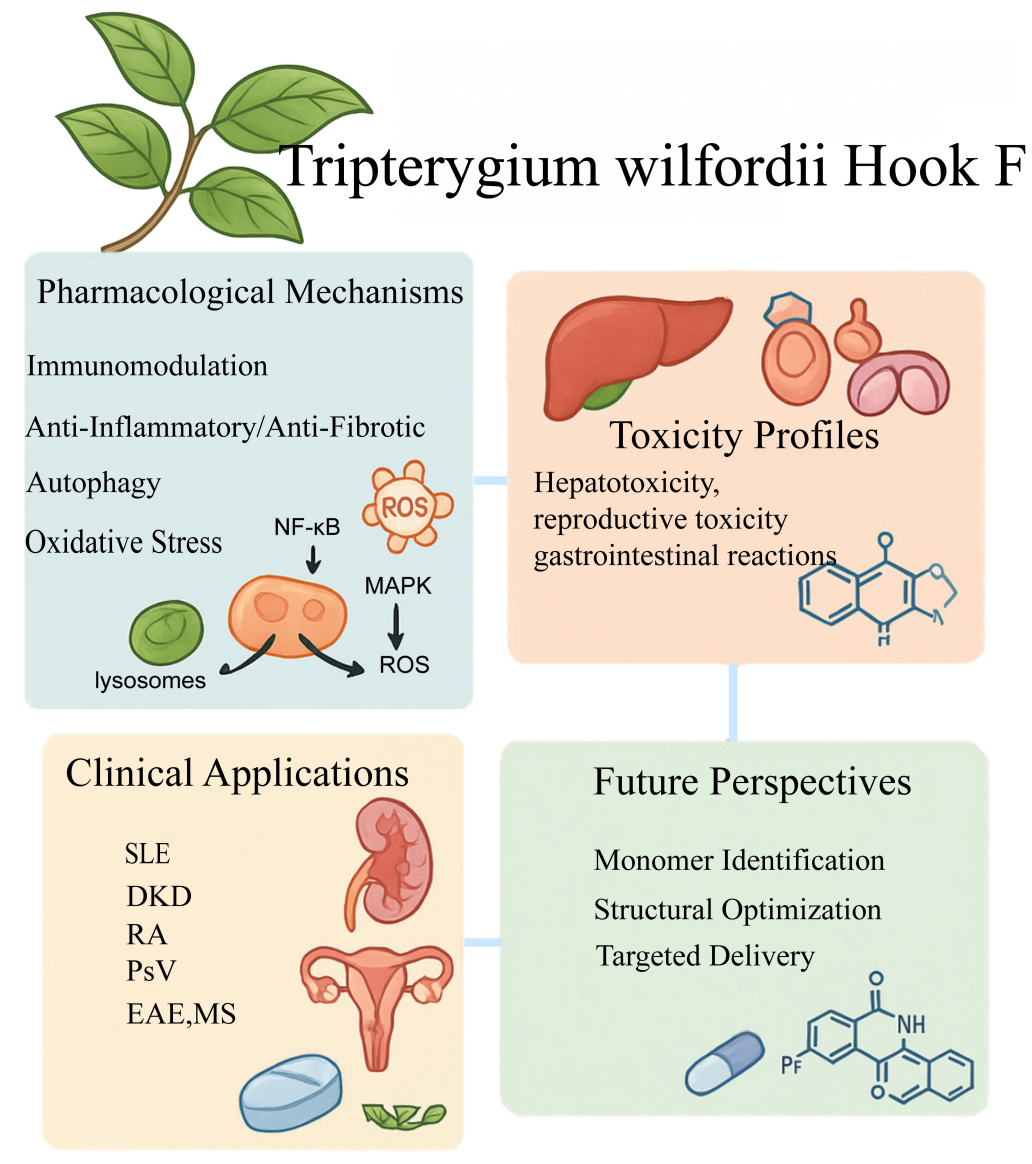
This figure summarizes the mechanism of action, clinical applications, safety issues and optimization strategies of Tripterygium glycosides. The drugs exert multi-target immunomodulatory and anti-inflammatory effects by targeting pathways such as NF-κB and MAPK, and can also regulate autophagy, fibrosis and oxidative stress. Clinical evidence supports their application in autoimmune diseases such as systemic lupus erythematosus, diabetic nephropathy, rheumatoid arthritis and psoriasis. However, their narrow therapeutic window, hepatotoxicity and reproductive toxicity and other safety issues limit their wide application. Structural optimization through the identification of active monomers (such as triptolide), prodrug delivery strategies and the development of safer derivatives (such as LLDT-8) can improve their therapeutic index and clinical translation potential.

TG exerts its therapeutic effects through multiple, interrelated pharmacological mechanisms. These include immune modulation, anti-inflammatory actions, anti-fibrotic effects, and the regulation of autophagy, apoptosis, and oxidative stress (Figure 1).

### 2.1 Immunomodulation and cytokine suppression

TG demonstrates potent immunosuppressive activity by attenuating excessive immune responses through multiple signaling cascades. These mechanisms collectively underpin TG's therapeutic efficacy in autoimmune disorders such as RA and SLE.

TG exerts significant anti-inflammatory effects by suppressing the NF-κB and MAPK signaling pathways. In macrophages and T lymphocytes, TG reduces the phosphorylation of IKK and IκBα, thereby inhibiting the nuclear translocation of NF-κB p65 and decreasing the transcription of pro-inflammatory cytokines, including TNF-α, IL-1β, and IL-6 (6). Additionally, TG inhibits the phosphorylation of MAPK family members, including ERK1/2 and p38, thereby attenuating AP-1 activation and further reducing inflammatory gene expression (7). In a diabetic nephropathy rat model, TG reduced glomerular macrophage infiltration and downregulated TNF-α, IL-1β, and TGF-β1 expression through suppression of the p38 MAPK and NF-κB signaling pathways. Emerging evidence suggests that TG also dampens the JAK/STAT pathway in T lymphocytes, thereby mitigating cytokine-driven proliferation. In collagen-induced arthritis models, TG and its monomer triptolide restored the Th17/Treg balance through inhibition of the JAK/PTEN–STAT3 axis, consequently suppressing IL-17 and IL-23 production.

TG promotes immune homeostasis by modulating T-cell differentiation and B-cell function. It enhances the frequency and suppressive capacity of regulatory T cells (Tregs) while inhibiting Th17 lineage commitment, thereby shifting the immune equilibrium toward a more tolerogenic state (8). In RA and SLE preclinical models, TG increased FoxP3 expression in CD4? T cells and reduced ROR γ t-driven Th17 responses, decreasing IL-17 release. Network pharmacology research supports TG's suppression of Th17 differentiation and IL-17 signaling in autoimmune skin disease models (9). TG also interferes with antigen presentation by inhibiting dendritic cell maturation and B-cell activation. TG extracts reduce the expression of dendritic cell co-stimulatory molecules (CD80 and CD86) and suppress cytokine production (e.g., IL-12 and IL-23), thereby impairing Th1/Th17 priming and attenuating antibody-mediated immune responses (10). These concerted immunoregulatory effects—namely, the suppression of pro-inflammatory signaling pathways, modulation of T- and B-cell balance, and inhibition of antigen presentation—form the mechanistic foundation for TG's clinical applications in autoimmune and inflammatory diseases (11).

### 2.2 Anti-inflammatory and anti-fibrotic effects

TG exhibits strong anti-inflammatory and anti-fibrotic properties across various disease models, including renal, hepatic, pulmonary, and dermal fibrosis.

In DKD models, TG markedly reduces proteinuria, glomerulosclerosis, and renal inflammation. Mechanistically, TG suppresses the activation of p38 MAPK and NF-κB signaling, thereby diminishing glomerular macrophage infiltration and downregulating the expression of pro-inflammatory cytokines (TNF-α, IL-1β) as well as the pro-fibrotic mediator TGF-β1 (2). Meta-analyses confirm a significant reduction in proteinuria and serum creatinine in diabetic nephropathy patients treated with TG compared to control groups (12). Moreover, in Adriamycin-induced nephropathy, TG suppresses activation of the TGF-β1/Smad signaling pathway, downregulates Smad2/3 phosphorylation, and reverses epithelial–mesenchymal transition (EMT), thereby alleviating glomerulosclerosis and tubulointerstitial fibrosis (13). TG also attenuates fibrosis through suppression of the TGF-β/Smad signaling pathway, a central regulator of fibrogenesis. In models of renal interstitial fibrosis, TG decreases TGF-β1 secretion, reduces Smad2/3 phosphorylation, and upregulates the inhibitory mediator Smad7, thereby dampening the pro-fibrotic transcriptional program. These molecular effects are accompanied by reduced deposition of collagen I/III and fibronectin within the renal interstitium (13, 14). Similar anti-fibrotic efficacy has been observed in fibrotic models of liver and skin, supporting TG's broad-spectrum anti-fibrotic potential.

TG restores autophagic flux—crucial for inhibiting fibrogenesis—particularly in kidney injury models. In diabetic nephropathy, triptolide (a TG monomer) reactivates autophagy through the PTEN/Akt/mTOR signaling axis, triggering reduction of extracellular matrix and fibrotic markers via miR-141-3p modulation (15). Activation of AMPK/mTOR by TG further enhances autophagy, supporting cellular homeostasis and reducing ECM accumulation. These autophagy-dependent effects are instrumental in mediating TG's anti-fibrosis action. TG exerts antioxidant effects by attenuating reactive oxygen species (ROS) and promoting endogenous antioxidant responses (e.g., Nrf2/HO-1). This antioxidant action synergizes with TGF-β/Smad pathway inhibition to effectively mitigate oxidative stress-induced fibrotic signaling, such as ERK and p38 MAPK activation, further enhancing tissue repair and reducing fibrosis progression (2, 15).

### 2.3 Autophagy regulation

TG, particularly its active constituent triptolide, exerts a profound regulatory effect on autophagy—a cellular process essential for maintaining homeostasis, degrading damaged organelles, and preventing fibrogenic transformation. Restoration of autophagy is a key mechanism underlying the therapeutic efficacy of TG in DKD, systemic inflammation, and fibrosis.

In DKD, impaired podocyte autophagy contributes to glomerular injury, proteinuria, and disease progression. TG restores autophagic flux by increasing the LC3-II/LC3-I ratio and decreasing p62 expression, molecular markers indicative of enhanced autophagy and lysosomal activity (16). These changes are associated with increased nephrin and podocin expression, improved cytoskeletal integrity, and reduced apoptosis in glomerular epithelial cells (17). The PTEN/Akt/mTOR signaling cascade plays a central role in the regulation of autophagy. Under hyperglycemic conditions, PTEN expression is suppressed, leading to overactivation of Akt and mTORC1 and subsequent inhibition of autophagy. TG counteracts this effect by upregulating PTEN, reducing Akt phosphorylation, and inhibiting mTORC1, thereby reactivating autophagy (15). *In vitro* and *in vivo* studies confirm that TG alleviates renal fibrosis and EMT through this pathway by promoting the degradation of fibrotic components and reducing TGF-β1 expression (15, 18).

In renal tubular epithelial cells, TG suppresses EMT by modulating the mTOR/Twist1 axis. Specifically, TG downregulates phosphorylated mTOR and Twist1, a key transcription factor that drives mesenchymal transition. This regulatory effect restores autophagic activity, reduces the expression of vimentin and α-SMA, and preserves epithelial markers such as E-cadherin (19). TG may also activate AMPK, a central energy sensor that antagonizes mTORC1. While the AMPK–mTOR interaction has been well established in the actions of other renoprotective agents, TG appears capable of engaging this pathway as well, particularly under metabolic stress conditions. This dual inhibition of mTOR signaling—through PTEN activation and AMPK engagement—enhances autophagic responsiveness and improves cellular adaptation to injury (20).

### 2.4 Oxidative stress and apoptosis

TG plays a dual role in regulating oxidative stress and apoptosis, two processes that are essential for maintaining cellular integrity and facilitating the removal of damaged or pathogenic cells. TG modulates intracellular ROS levels, activates endogenous antioxidant pathways, and selectively induces apoptosis in cells that are damaged or inflamed.

TG exerts antioxidative activity by attenuating excessive ROS production while enhancing cellular antioxidant defenses (21). However, studies show that triptolide—the principal active component of TG—can suppress the expression of Nrf2 and its downstream targets HO-1 and NQO1 in models exposed to both LPS and triptolide, thereby exacerbating oxidative injury in zebrafish liver. These findings suggest a context- and dose-dependent effect of TG on redox balance (22). Consistently, TG-induced hepatotoxicity in mice has been associated with downregulation of Nrf2/HO-1 and Bcl-2, along with increased Bax expression and lipid peroxidation, indicating that TG can reduce oxidative stress at therapeutic doses but may promote oxidative injury at higher or non-physiological concentrations (23). TG promotes apoptosis in pathogenic or stress-affected cells through mitochondrial- and ER-mediated pathways. Triptolide induces mitochondrial outer membrane permeabilization (MOMP), leading to cytochrome c release and activation of caspase-3 and caspase-9, thereby shifting the Bax/Bcl-2 balance toward apoptosis in immune and renal cell (24). Furthermore, TG aggravates ER stress by increasing ROS production and disrupting redox homeostasis, thereby activating unfolded protein response (UPR) pathways and inducing CHOP-mediated caspase cascades in models such as osteosarcoma cells and Sertoli cells.

TG-induced oxidative stress robustly activates MAPK family members—particularly JNK and p38—ultimately leading to mitochondrial dysfunction and apoptotic signaling. For example, ROS accumulation enhances JNK phosphorylation, which in turn promotes mitochondrial pore formation, cytochrome c release, and downstream caspase activation in reproductive and hepatic cell models (25). TG's pro-apoptotic activity exhibits notable selectivity: malignant cells, activated immune cells (e.g., dendritic cells), and fibrotic cells are considerably more susceptible than their healthy counterparts. This selective cytotoxicity is attributed to their intrinsically higher basal levels of ROS and ER stress, which render these cells more vulnerable to TG-induced oxidative perturbation and mitochondrial collapse (26).

### 2.5 Network pharmacology

Network pharmacology offers a systems-level perspective on how TG and TwHF-derived compounds interact with multiple molecular targets across diverse signaling pathways, thereby elucidating their multifaceted therapeutic effects in inflammatory, fibrotic, and autoimmune diseases.

A network pharmacology analysis evaluating TwHF in DKD identified 68 overlapping targets between TG bioactive constituents and DKD-associated genes. Key hub targets included PTGS2, RELA (NF-κB p65), AKT1, and MAPK8 (JNK). Enrichment analysis further highlighted several pivotal pathways—such as AGE–RAGE, IL-17, TNF, and insulin resistance—aligning well with the established multi-pathway regulatory actions of TG (27). In membranous nephropathy (MN), network pharmacology models identified 126 overlapping gene targets, implicating key pathways such as AGE–RAGE signaling, lipid metabolism, IL-17, and NF-κB—closely mirroring the immuno-inflammatory axes observed in DKD. Molecular docking analyses further demonstrated strong binding affinities between TG constituents and core targets such as PTGS2 and RELA, with docking energies below −5 kJ/mol, thereby reinforcing the relevance of these pathways in mediating TG's renoprotective effects (28). In SLE, network pharmacology analyses identified TG-mediated regulation of inflammatory and fibrotic signaling pathways, including NF-κB, STAT1/2, and Th17-related targets. Enrichment analysis highlighted pathways such as cytokine–cytokine receptor interaction and apoptosis. Key hub targets—including JAK1/2, STAT3, and TLR4—were also identified, consistent with the established immunomodulatory and anti-inflammatory effects of TG in experimental SLE models..

Investigations into Tripterygium hypoglaucum (a closely related species) have revealed overlapping molecular targets associated with both therapeutic efficacy—such as STAT3, VEGFA, and MMP9—and toxicity, including TP53 and components of the p53 signaling pathway. Network pharmacology analyses further highlighted the PI3K–Akt and p53 pathways as critical intersections between beneficial and adverse effects (29). These findings underscore the need to refine TG formulations to preserve therapeutic potency while minimizing toxicity risks, particularly hepatotoxicity and reproductive injury. Combined transcriptomic and metabolomic analyses of TwHF in DKD have validated predictions from network pharmacology, demonstrating that TG modulates key pathways including AGE–RAGE, TNF, IL-17, and insulin resistance. Molecular docking further confirmed strong binding affinities of TG constituents—such as kaempferol and triptolide—to core targets including PTGS2 and AKT1 (30). Together, these findings establish a robust mechanistic framework that integrates computational prediction with experimental validation.

## 3 Clinical applications

**Figure 2:**
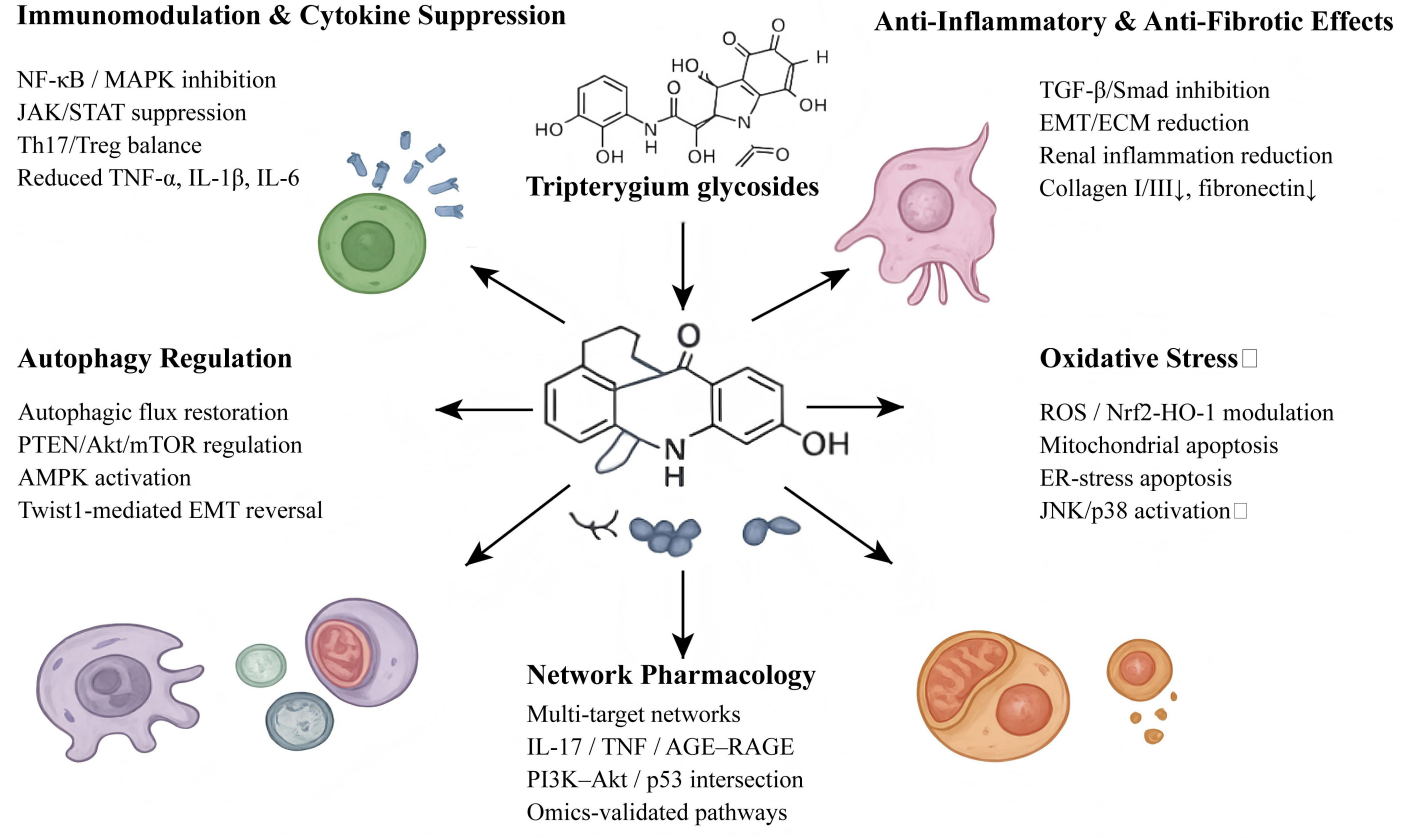
Multi-target mechanisms of Tripterygium glycosides (TG) in immune and metabolic diseases. TG exert therapeutic effects by inhibiting key inflammatory pathways (NF-κB, MAPK, and JAK/STAT), modulating immune cell balance, and restoring autophagy via PTEN/Akt/mTOR and AMPK signaling. TG also suppresses TGF-β/Smad-mediated fibrosis and oxidative stress, and induces selective apoptosis through mitochondrial and endoplasmic reticulum (ER) stress pathways. Network pharmacology highlights the multi-target interactions of TG in diseases such as diabetic kidney disease and systemic lupus erythematosus, supporting its broad therapeutic potential.

TG has been extensively evaluated in clinical settings, demonstrating therapeutic efficacy across autoimmune, renal, dermatologic, and ophthalmologic disorders. Below is a refined overview organized by disease domain, with emphasis on evidence from randomized controlled trials (RCTs) and meta-analyses (Figure 2).

### 3.1 Systemic lupus erythematosus

TG have shown promising efficacy as an adjunctive treatment in SLE, particularly in reducing disease activity and immunosuppressant dependence. A meta-analysis including eight RCTs with 538 SLE patients found that TG combined with conventional therapy significantly reduced disease activity (mean difference in SLEDAI = −1.66 [95% CI −2.07 to −1.26]) and improved overall response rate (RR = 1.21 [95% CI 1.11–1.32]) compared to conventional therapy alone (31). Importantly, the incidence of adverse events—including gastrointestinal discomfort and mild elevations in liver enzymes—did not differ significantly between TG and control groups, supporting its short-term safety and tolerability. Additional systematic reviews and real-world clinical studies have further corroborated these findings (31, 32). TG not only improves serologic markers—such as reducing anti-dsDNA antibodies and increasing complement C3 and C4 levels—but also shows potential glucocorticoid-sparing effects and may help reduce the risk of lupus flares as disease activity decreases (31).

Natural compounds primarily ameliorate SLE by inhibiting NF-κB and JAK/STAT signaling, restoring Treg/Th17 balance, and reducing autoantibody production, thereby attenuating systemic inflammation. Recent mechanistic investigations have revealed that triptolide, the major active constituent of TG, modulates immune dysregulation in SLE through several convergent pathways (31). Triptolide corrects the abnormal distribution of Th and Tc effector T-cell subsets observed in lupus models, thereby mitigating dysfunctional T-cell skewing. Within germinal center–related immunity, triptolide restores the balance between follicular regulatory T cells (Tfr) and follicular helper T cells (Tfh) (33), effectively limiting excessive B-cell help, restraining aberrant germinal center activation, and reducing autoantibody production. At the B-cell–intrinsic level, triptolide upregulates miR-146a, a key negative regulator of the TLR7 signaling pathway, thereby attenuating TLR7-driven B-cell hyperactivation and reducing the generation of pathogenic autoantibodies (34).

Given its dual impact on inflammation and immune dysregulation, TG is increasingly regarded as a potential steroid-sparing agent in lupus management. Nevertheless, long-term safety evaluations and large-scale randomized controlled trials conducted outside of East Asia are still required to substantiate these findings and facilitate broader regulatory acceptance worldwide.

### 3.2 Diabetic kidney disease

Many natural compounds have demonstrated renoprotective effects in DKD by acting on several convergent molecular pathways. Recent evidence shows that these agents commonly inhibit NF-κB–mediated inflammation, suppress TGF-β/Smad-driven fibrotic signaling (35, 36), reduce renal macrophage infiltration, and restore impaired podocyte autophagy—mechanisms that align closely with key pathological drivers of DKD progression (37–39). Against this mechanistic background, TG have gained increasing attention as a complementary therapy in DKD, particularly in patients with persistent proteinuria despite standard RAAS blockade. A randomized, controlled clinical trial in DKD demonstrated that *Tripterygium wilfordii* extracts significantly reduced 24-hour urinary protein and improved overall clinical response compared with conventional treatment alone (40). Consistent findings were observed in a meta-analysis of stage IV DKD, where TG therapy further decreased proteinuria and increased serum albumin levels while maintaining an acceptable safety profile (41). Together, these clinical data support the adjunctive use of TG in proteinuric DKD, especially in patients who show suboptimal response to RAAS inhibition.

Mechanistically, recent studies have shown that triptolide confers renoprotection in DKD by modulating microRNA-dependent pathways that govern autophagy and fibrotic progression. Triptolide restores impaired autophagy in diabetic kidneys by suppressing miR-141-3p, thereby releasing PTEN from microRNA-mediated inhibition and subsequently downregulating the Akt/mTOR signaling cascade, a key suppressor of autophagic activity. Reestablishment of PTEN–Akt/mTOR signaling improves autophagic flux and ultimately reduces renal fibrotic injury in DKD models (15). In parallel, triptolide attenuates tubular EMT—a central mechanism driving tubulointerstitial fibrosis—through inhibition of miR-188-5p, leading to suppression of the PI3K/Akt pathway and downstream mesenchymal markers such as α-SMA and vimentin, while preserving epithelial characteristics (42). Taken together, these findings suggest that triptolide alleviates DKD by simultaneously enhancing autophagy and suppressing EMT through two distinct microRNA–Akt regulatory axes.

From a safety perspective, adverse events such as gastrointestinal discomfort, transient hepatotoxicity, and menstrual disturbances were more common in TG-treated groups, although serious toxicity was rare (32). Although no standardized safety threshold has been established, clinical experience and available trials suggest that lower daily doses and limiting treatment duration may help reduce TG-related adverse events (43). Given the unmet need for effective anti-proteinuric therapies beyond SGLT2 inhibitors and GLP-1 receptor agonists, TG represents a promising adjunctive option, particularly in resource-limited settings or among patients who are intolerant of standard treatments. Nevertheless, long-term randomized controlled trials with robust renal endpoints (e.g., eGFR slope, progression to dialysis) and comprehensive real-world safety data are urgently required to define TG's therapeutic role in global DKD management.

### 3.3 Rheumatoid arthritis

RA is a chronic autoimmune disorder characterized by persistent synovial inflammation and progressive joint damage (44–46). Current management emphasizes early diagnosis and timely initiation of disease-modifying anti-rheumatic drugs (DMARDs) to prevent structural deterioration. Standard treatment follows a stepwise strategy, starting with conventional synthetic DMARDs such as methotrexate, leflunomide, sulfasalazine, and hydroxychloroquine (47). Patients with inadequate response are advised to escalate to biologic DMARDs targeting TNF-α, IL-6R, or CTLA-4-Ig, or to targeted synthetic DMARDs such as Janus kinase inhibitors, to achieve optimal disease control (48).

In parallel, TCM has been widely incorporated into RA management in China, particularly for patients with chronic pain, intolerance to immunosuppressive therapy, or preference for integrative medicine. TG have gradually become a cornerstone herbal therapy in the clinical management of RA in China and are increasingly recognized for their potent anti-inflammatory and immunomodulatory properties. Multiple meta-analyses demonstrate that TG is non-inferior to MTX—the first-line conventional DMARD—in improving key clinical endpoints such as joint swelling, morning stiffness, pain scores, and erythrocyte sedimentation rate (ESR) (49). A meta-analysis including 11 randomized controlled trials with 1,675 RA patients evaluated *Tripterygium wilfordii* preparations and showed that they increased overall clinical effectiveness by approximately 20% and reduced the risk of adverse events by about 18%, with markedly greater improvements in ESR, RF, and CRP compared with control treatments (50). Moreover, combining TG with MTX or leflunomide produces synergistic therapeutic benefits without markedly increasing adverse events, supporting TG as an effective adjunctive strategy for moderate to severe RA (51).

In RA, natural compounds generally suppress NF-κB, MAPK, and JAK/STAT pathways, reduce IL-1β, IL-6, and TNF-α levels, and inhibit fibroblast-like synoviocyte proliferation, leading to improved synovial inflammation and joint protection (52, 53). Mechanistically, Tripterygium-derived compounds suppress RA inflammation primarily by inhibiting the IL-17/NF-κB signaling pathway, leading to marked reductions in IL-17, TNF-α, IL-6, and IL-1β levels in synovial tissues. They also restrain the pathological proliferation of fibroblast-like synoviocytes by downregulating CDK2 and restoring p21, while promoting apoptosis through increased caspase-3 and reduced Bcl-2/Bax ratios. Together, these actions correct synovial hyperplasia and help mitigate joint inflammation and damage (50, 54). Despite its therapeutic benefits, TG is limited by dose-related gastrointestinal discomfort, menstrual irregularities, leukopenia, and hepatotoxicity. Systematic reviews indicate that short-term regimens of approximately 1 mg/kg/day (not exceeding 1.5 mg/kg/day, divided into three post-meal doses) generally show acceptable safety, whereas higher doses or prolonged use markedly increase toxicity. Chinese expert consensus therefore positions TG as an adjunctive or alternative csDMARD for patients intolerant of, or unresponsive to, conventional DMARDs (55). However, most clinical studies originate from China, and the lack of high-quality international trials continues to restrict TG's incorporation into Western treatment guidelines.

### 3.4 Psoriasis and skin disorders

TG has shown therapeutic benefit in moderate-to-severe plaque psoriasis (PsV), offering a potential alternative or adjunct to agents such as acitretin and cyclosporine. In a randomized trial enrolling 115 PsV patients treated for 8 weeks, both TG and acitretin significantly reduced PASI scores, but TG demonstrated a better safety profile, with fewer mucocutaneous reactions, lipid abnormalities, and liver enzyme elevations. Although not statistically significant, a higher proportion of patients achieved PASI-50 with TG than with acitretin (50% vs. 43%) (56).

Most natural compounds improve psoriasis by inhibiting the IL-23/IL-17 axis and NF-κB/MAPK signaling, thereby reducing Th17-mediated inflammation and normalizing keratinocyte proliferation (57, 58). Further evidence from combination therapy trials suggests that TG may exert synergistic effects when used with acitretin. In one study involving 36 patients, the combination of TG and acitretin led to superior PASI score reductions and more stable liver function parameters compared to acitretin monotherapy (59). Although a subsequent retraction raised concerns regarding data integrity, the hypothesis of additive benefit remains plausible and is supported by TG's known mechanisms of action. Mechanistically, TG suppresses Th17 cell differentiation, inhibits IL-17 and TNF-α signaling, and downregulates dendritic cell activation—pathways central to psoriatic inflammation (9). Triptolide, a major active constituent of TG, has also been shown to promote Treg cell differentiation, further contributing to immune homeostasis in psoriatic lesions (60).

Short-term TG therapy ( ≤ 8 to 12 weeks) in psoriasis is generally well tolerated, with adverse events mainly limited to mild gastrointestinal discomfort and reversible menstrual irregularities. No severe hepatotoxicity or nephrotoxicity has been reported under controlled dosing conditions. However, broader dermatologic use remains limited by the lack of large, multicenter RCTs, uncertainties regarding long-term safety, and batch-to-batch variability in TG preparations. Consequently, further high-quality evidence is required before TG can be routinely incorporated into international dermatologic practice.

### 3.5 Ophthalmologic and neuroinflammatory disorders

Emerging clinical and preclinical evidence suggests that TG may offer therapeutic benefit in ocular autoimmune and neuroinflammatory diseases, particularly in Graves' orbitopathy (GO) (61, 62) and experimental autoimmune encephalomyelitis (EAE), a model for multiple sclerosis (MS) (1).

Growing evidence from systematic reviews and meta-analyses indicates that TG may provide clinical benefit in patients with active thyroid-associated orbitopathy (TAO). A comprehensive meta-analysis of Chinese cohorts reported that TG, either as monotherapy or in combination with low-dose glucocorticoids, was associated with significant reductions in clinical activity score (CAS), improvements in soft-tissue inflammation, and, in some studies, modest improvements in proptosis and extraocular muscle involvement. Another systematic review similarly concluded that TG-based regimens improved overall clinical response rates and disease inactivation compared with conventional therapies (61, 62). These findings indicate that TG may serve as a corticosteroid-sparing agent in GO, providing a well-tolerated oral option for patients with contraindications to IVMP or concerns about steroid-related toxicity.

Natural compounds mitigate neuroinflammation by suppressing microglial activation and NF-κB/MAPK pathways, reducing IL-17A and TNF-α production, and improving blood–brain barrier integrity (53). In the realm of neuroinflammatory diseases, triptolide—the primary active component of TG—has demonstrated efficacy in attenuating neuroinflammation in EAE models. Mechanistic studies indicate that triptolide reduces BBB permeability, suppresses microglial activation, and downregulates pro-inflammatory cytokines such as IL-17A and TNF-α within the central nervous system (63). Additionally, experimental evidence demonstrates that triptolide suppresses Th17 cell infiltration while enhancing regulatory T cell (Treg) proliferation within the spinal cord microenvironment, suggesting its potential in limiting demyelination and neurodegeneration (53, 64). While these preclinical data are promising, clinical trials of TG or triptolide in multiple sclerosis or other neuroimmune disorders remain lacking, and concerns about CNS toxicity must be thoroughly addressed.

Although data in these indications are limited, the targeted immunosuppressive profile of TG, along with its ability to modulate T-cell responses and cytokine networks, provides a rationale for further investigation in ophthalmic and neuroinflammatory settings. Well-designed clinical trials and mechanistic translational research are required to determine optimal dosing parameters, safety profiles, and CNS penetration of TG in human subjects.

## 4 Toxicity profiles, safety challenges, and barriers to clinical translation

**Figure 3:**
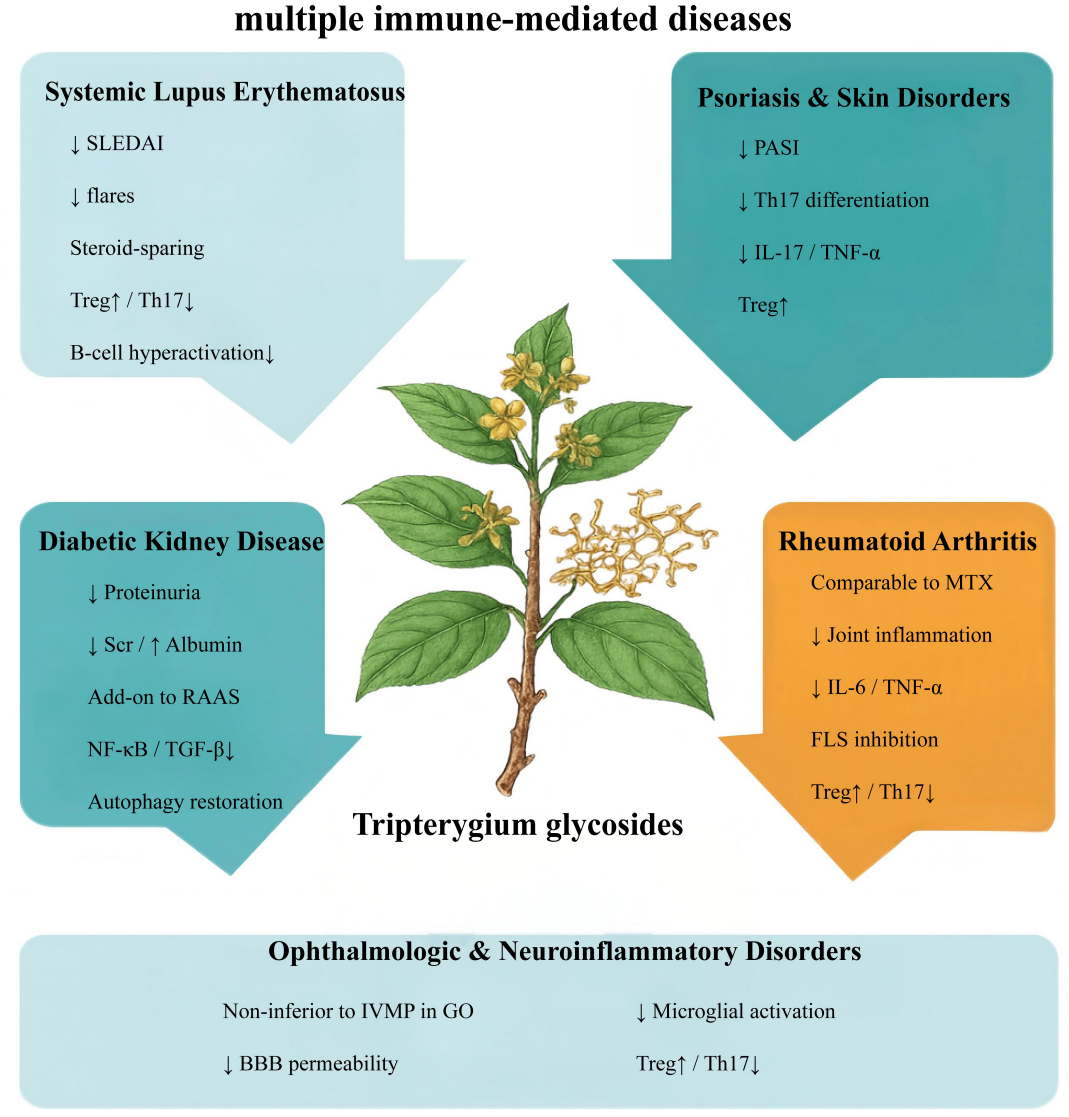
The disease-specific mechanisms of Tripterygium glycosides. In systemic lupus erythematosus, TG inhibits NF-κB and JAK/STAT signaling and restores Treg/Th17 balance; in diabetic kidney disease, it suppresses TGF-β/Smad signaling, reduces macrophage infiltration, and restores podocyte autophagy; in rheumatoid arthritis, TG downregulates IL-6, IL-1β, and TNF-α to alleviate synovial inflammation; in psoriasis, it inhibits Th17 differentiation and IL-17/TNF-α signaling; and in neuroinflammatory disorders, TG reduces microglial activation and improves blood–brain barrier integrity.

TG possess strong therapeutic potential across multiple immune-mediated and inflammatory diseases; however, their clinical utility is constrained by a narrow safety margin and notable systemic toxicities. These challenges have prompted intensive research efforts focused on three major directions: mitigating inherent toxicities, refining molecular structures to improve the therapeutic window, and developing advanced delivery systems to enhance targeted distribution. This section synthesizes current progress in toxicity reduction strategies and the development of safer TG-derived compounds, providing a foundation for their broader clinical translation (Figure 3; Table 1).

**Table 1:** Comparative toxicity profiles, mechanistic features, and therapeutic windows of triptolide, LLDT-8, and triptonide.

**Feature**	**Triptolide (parent diterpenoid)**	**LLDT-8 (5R-5-hydroxytriptolide)**	**Triptonide (C14 epimer; lacks epoxide)**
Overall toxicity level	High	Moderate–Low	Low
Therapeutic window	Narrowest; toxicity close to effective dose	Wider than triptolide	Widest among known derivatives
Hepatotoxicity	Significant hepatocellular injury; ROS accumulation; mitochondrial depolarization	Reduced mitochondrial stress and lower ALT/AST elevation	Minimal hepatocellular toxicity; greatly reduced ROS burden
Reproductive toxicity	High; Sertoli cell apoptosis; impaired spermatogenesis; menstrual suppression	Markedly reduced Sertoli cell toxicity; improved reproductive safety	Lowest reproductive toxicity; minimal impact on sperm morphology/function
Hematologic toxicity	Leukopenia and bone-marrow suppression at therapeutic doses	Low hematologic toxicity in preclinical studies	Very low; limited bone marrow impact
Gastrointestinal toxicity	Frequent nausea, diarrhea; dose-dependent	Mild; significantly attenuated	Mild; lower incidence compared with triptolide
Mechanistic basis of toxicity	Non-selective transcription inhibition; mitochondrial damage; excessive ROS; apoptosis induction	Preserves immune modulation but reduces mitochondrial injury and apoptosis signaling	Lack of C14 epoxide reduces covalent reactivity; lower affinity for toxicity-related off-targets
Immunomodulatory potency	Very strong	Comparable or superior to triptolide in some models	Strong immunomodulatory effects with better tolerability
Structural features driving toxicity	C14 epoxide ring strongly associated with hepatotoxicity and reproductive toxicity	Hydroxylation at C5 reduces cytotoxicity	Loss of epoxide at C14 and epimerization markedly reduce toxicity
Clinical development status	Parent compound; limited by toxicity; no global approvals	Phase I/II trials (lupus nephritis, inflammatory myopathy)	Preclinical/early translational studies; high potential
Overall safety profile	Poorest	Improved; clinically manageable	Best among TG derivatives studied to date

This table system compares the key features of three representative tripterygium diterpenoids (triptolide, LLDT-8, and celastrol) in terms of toxicity, safety margin, and immunomodulatory efficacy. Triptolide, as the parent compound, has strong immunomodulatory activity, but its C14 epoxide structure is closely related to significant liver toxicity, reproductive toxicity, and bone marrow suppression, resulting in the narrowest therapeutic window. LLDT-8, by introducing a hydroxyl group at the C5 position, maintains or enhances immunomodulatory efficacy while significantly reducing mitochondrial damage and apoptotic signals, demonstrating a wider therapeutic window and clinically manageable safety, and has entered phase II clinical trials.

### 4.1 Toxicity and clinical toxicity-related failures

Despite its proven therapeutic efficacy, TG are associated with a narrow therapeutic window and a well-recognized spectrum of dose- and time-dependent toxicities. The most frequently reported adverse effects include gastrointestinal symptoms (nausea, diarrhea, abdominal discomfort), menstrual irregularities, hepatic dysfunction, leukopenia, and reproductive toxicity. In pharmacovigilance studies and real-world cohort analyses, the incidence of adverse events varies between 20–40% (65), but serious toxicity remains relatively rare when TG is administered at ≤ 1.5 mg/kg/day for a treatment duration under 3–6 months (43, 66). Hepatotoxicity is usually transient and reversible but warrants regular monitoring of liver enzymes (ALT, AST). Hematologic abnormalities, such as leukopenia or thrombocytopenia, may occur and necessitate temporary drug withdrawal or dose reduction. Reproductive toxicity—including oligospermia and menstrual suppression—is dose-related and often reversible upon discontinuation but remains a major concern in younger patients or those seeking fertility (32).

Nevertheless, these toxicities have led to clinically meaningful treatment discontinuation, particularly in real-world cohorts and randomized trials. In SLE and RA studies, gastrointestinal reactions, elevated liver enzymes, and leukopenia were among the leading causes of early withdrawal, while dose-related reproductive toxicity—such as oligospermia and menstrual suppression—prompted discontinuation in younger patients or those concerned about fertility (50, 66). Beyond routine clinical practice, toxicity has also contributed to translational failures of TG-derived monomers. Multiple early-phase international development attempts involving triptolide were terminated due to severe gastrointestinal injury, hepatocellular toxicity, or bone-marrow suppression, preventing advancement into late-stage trials. In addition, batch-to-batch variability in TG preparations has resulted in inconsistent toxicity profiles, further limiting global adoption (67–69). Collectively, these toxicity-related clinical and translational failures highlight the need for standardized formulations, stringent monitoring, and the development of safer TG derivatives.

### 4.2 Challenges in translating preclinical toxicity to clinical settings

Although animal studies provide essential insights into the organ-specific and dose-dependent toxicity of TG and triptolide, several challenges significantly limit the direct translation of these findings to clinical practice. First, species differences in metabolic pathways profoundly influence the bioactivation and clearance of diterpenoids. Rodents metabolize triptolide through hepatic CYP isoforms that differ from those predominant in humans, leading to discrepancies in systemic exposure and hepatotoxicity thresholds (70). Second, dose–exposure relationships vary substantially between animals and humans. Many preclinical toxicity studies employ supratherapeutic or bolus doses to demonstrate organ injury, whereas clinical dosing is typically lower, fractionated, and influenced by interindividual variability, comorbidities, and concomitant therapies (71). Third, animal models often fail to recapitulate human susceptibility factors, such as pre-existing liver disease, reproductive hormonal fluctuations, gut microbiota–mediated drug metabolism, and genetic polymorphisms in detoxification enzymes—all of which modulate TG toxicity in real-world populations (72). Additionally, the composition of TG preparations used in animal experiments may not match clinical formulations. Variability in extraction methods, triptolide content, and the presence of other diterpenoids contributes to inconsistent toxicity patterns that may not faithfully represent human exposure. Finally, animal studies typically have short observation windows and cannot fully capture chronic toxicity, cumulative reproductive impairment, or long-term immunosuppression, which are clinically relevant concerns in autoimmune diseases (68). Collectively, these translational gaps underscore the need for rigorous pharmacokinetic bridging, standardized formulations, and carefully monitored clinical dosing strategies to safely apply preclinical toxicity data to human therapy.

### 4.3 Comparative toxicity profiles and the therapeutic window problem

Tripterygium-derived monomers differ substantially in their toxicity profiles, therapeutic indices, and organ-specific adverse effects (68). Triptolide, the most potent bioactive constituent of TG, also exhibits the narrowest therapeutic window, with dose-dependent hepatic, reproductive, and hematologic toxicities. In contrast, next-generation derivatives such as LLDT-8 (5R-5-hydroxytriptolide) and triptonide demonstrate expanded safety margins owing to structural modifications that attenuate off-target cytotoxicity while preserving key immunomodulatory activities.

Triptolide serves as the reference point for toxicity comparison (73). As the primary driver of TG-related adverse events, triptolide exerts potent inhibition of transcriptional processes and mitochondrial function. These actions lead to hepatocellular injury (via excessive ROS production, mitochondrial depolarization, and impaired autophagy flux), reproductive toxicity (Sertoli cell apoptosis, disrupted spermatogenesis, menstrual suppression), and hematologic suppression (leukopenia and bone-marrow inhibition) (74, 75). This constellation of toxicities defines the pharmacologic constraints that newer derivatives aim to overcome.

LLDT-8 (5R-5-hydroxytriptolide) represents one of the most successful attempts to mitigate triptolide-associated toxicity (76). Preclinical evidence indicates that LLDT-8 achieves comparable or even superior immunomodulatory efficacy in SLEand RA models (77), yet exhibits markedly reduced cytotoxicity toward hepatocytes and Sertoli cells (78). Mechanistically, LLDT-8 diminishes mitochondrial ROS accumulation and stabilizes mitochondrial membrane potential, leading to lower hepatotoxic and reproductive risk. Importantly, LLDT-8 retains inhibition of NF-κB and STAT3 signaling while promoting Treg expansion with less activation of apoptotic pathways (79), which has enabled its progression into phase I/II clinical trials for lupus nephritis and idiopathic inflammatory myopathy (67).

Triptonide, a naturally occurring C14 epimer lacking triptolide's epoxide moiety (67), demonstrates an even more favorable safety profile (80). Elimination of the C14 epoxide—recognized as a key structural determinant of hepatic and reproductive toxicity—significantly attenuates triptonide's impact on liver function and fertility (67). Comparative toxicology studies show that triptonide induces substantially less Sertoli cell apoptosis, exhibits lower IC50 hepatocyte toxicity, and causes fewer sperm abnormalities than triptolide (67, 81). Despite this improved tolerability, triptonide maintains potent immunoregulatory and anti-inflammatory properties. SAR analyses further confirm that modifications at the epoxide, C14 hydroxyl, and lactone ring positions critically influence the balance between efficacy and toxicity (67).

Collectively, these comparative data define a clear toxicity hierarchy—triptolide > LLDT-8 > triptonide—illustrating how rational molecular modification can markedly expand the therapeutic window of TG-derived diterpenoids. Continued comparative toxicology and mechanistic profiling will be essential for identifying the most promising candidates for clinical translation and for guiding the development of safer, next-generation TG-based therapeutics.

### 4.4 Regulatory barriers and gaps limiting global acceptance

Despite promising therapeutic potential, TG and their derivatives face significant regulatory barriers that limit global acceptance. The lack of standardized extraction processes and batch-to-batch consistency remains a major obstacle, as variability in triptolide content directly affects both efficacy and toxicity (32, 65). Furthermore, toxicology data for TG extracts are often heterogeneous, derived from mixed formulations, or insufficiently aligned with modern regulatory expectations for purity-defined small molecules. The absence of internationally harmonized quality-control frameworks, combined with incomplete long-term safety data and limited Phase II/III trial evidence, has prevented broader approval outside China. Concerns regarding reproductive toxicity, hepatotoxicity, and dose–response variability further contribute to regulatory hesitancy (32, 74), especially in regions with stringent pharmacovigilance requirements. Additionally, the incomplete mechanistic elucidation of off-target toxicities and the lack of validated biomarkers for monitoring TG-induced adverse effects constitute remaining gaps (32). Addressing these issues through standardization, rigorous toxicology profiling, and globally compliant clinical development pathways will be essential for advancing TG-based therapeutics toward wider international adoption.

## 5 Future perspectives and global translation of TG-based therapeutics

Future development of TG-based therapeutics requires high-quality mechanistic and clinical evidence, standardized safety management, and globally harmonized quality control. Although TG show broad immunomodulatory and antifibrotic potential, their translation is limited by intrinsic diterpenoid toxicity and formulation variability. Advances in monomer identification, structural optimization, and targeted delivery—together with emerging technologies such as AI-assisted design and biosynthetic engineering—are paving the way for safer, more precise next-generation TG derivatives. These innovations collectively outline a clearer path toward global adoption of TG-based therapies.

### 5.1 Novelty and limitations of this review

This review provides an integrated and cross-disciplinary perspective on TG by systematically linking their multi-target pharmacological mechanisms with clinical applications across autoimmune, renal, dermatologic, and neuroinflammatory diseases. Unlike previous reviews that typically focus on a single disease or on triptolide alone, our work offers several novel contributions. First, we comparatively analyze disease-specific mechanistic pathways and highlight areas of convergence—such as NF-κB, JAK/STAT, TGF-β/Smad, and autophagy regulation—that collectively explain TG's broad therapeutic spectrum. Second, we provide one of the most detailed summaries to date of toxicity-related failures, derivative optimization strategies, and structure–toxicity relationships, including comparative toxicity profiles of next-generation monomers such as LLDT-8 and triptonide. Third, we outline a forward-looking framework for TG standardization, monomer differentiation, and structural refinement that may inform future drug-development pipelines.

Nevertheless, this review has inherent limitations. Evidence supporting TG efficacy and safety remains heterogeneous, with most randomized trials being small, single-center, or conducted exclusively in Chinese populations. Mechanistic studies across diseases vary in depth, and some conclusions rely on preclinical data that may not fully translate to humans. Additionally, long-term safety data, standardized toxicology protocols, and globally harmonized quality-control criteria for TG preparations are insufficient, limiting definitive assessment of therapeutic windows and regulatory feasibility. These gaps highlight the need for large, multi-ethnic clinical trials, rigorous pharmacokinetic and toxicology profiling, and international consensus on quality standards to advance TG-based therapeutics toward global acceptance.

### 5.2 Clinical risk-mitigation strategies and safety management

To mitigate these risks, several risk management strategies have been proposed and implemented in clinical practice. First, individualized dosing and duration control are essential. Most guidelines recommend limiting the cumulative dose and total course duration, especially in combination regimens. Baseline assessments (e.g., liver/renal function, complete blood count, pregnancy testing) and periodic monitoring (every 2–4 weeks during the initial phase) are advised to detect early signs of toxicity (55). Second, co-administration with hepatoprotective agents or leukopoiesis-supportive therapies (e.g., leucogen) has been explored with partial success in observational studies (65). Third, the development of TG monomer derivatives with improved selectivity and reduced toxicity profiles is actively ongoing and may represent the next generation of TG-based therapeutics (67). Furthermore, the standardization of TG extracts and quality control across manufacturers are critical for ensuring consistent bioavailability and minimizing unexpected toxicity due to formulation variability (82). Lastly, patient education on early warning signs (e.g., fatigue, bleeding, jaundice, menstrual delay) is an integral part of safe TG administration.

While clinical risk-mitigation strategies can reduce the incidence of adverse reactions during TG therapy, they do not fundamentally address the intrinsic toxicity of major diterpenoids such as triptolide. Therefore, substantial efforts have been directed toward optimizing molecular structures and developing next-generation TG derivatives to overcome the narrow therapeutic window (Figure 3).

### 5.3 Technology-driven innovation, regulatory challenges, and global translation

**Figure 4:**
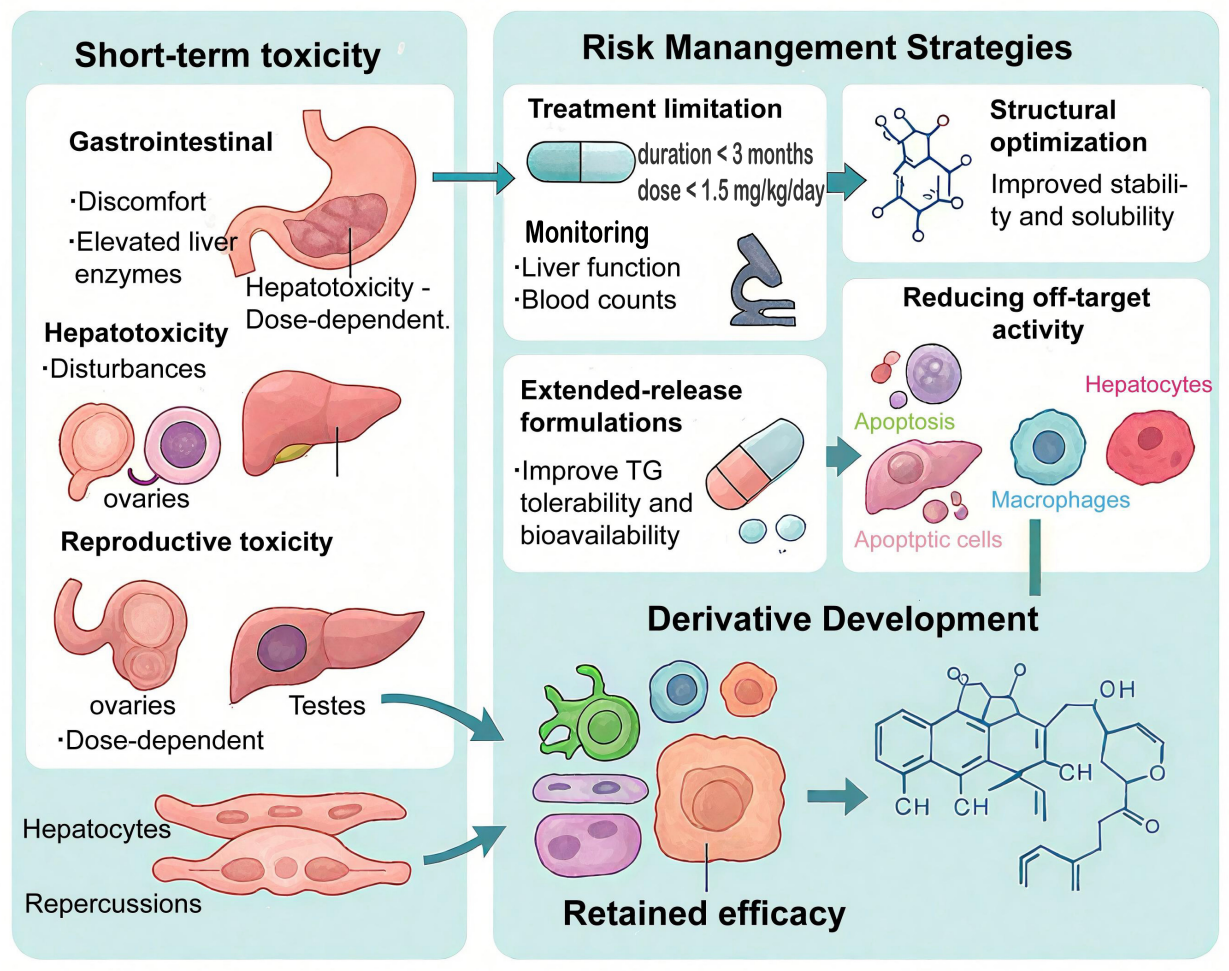
Toxicity, risk management, and derivative development of TG. This figure highlights TG's main toxicities—gastrointestinal, hepatic, and reproductive—and strategies to improve safety, including short-term use, monitoring, and extended-release formulations. Structural optimization and derivative development focus on reducing toxicity while preserving efficacy.

To overcome the narrow therapeutic window of TG, recent research has focused on a multi-tiered safety-optimization framework that integrates monomer identification, structural optimization, prodrug engineering, and targeted delivery. These methods collectively aim to retain TG's potent anti-inflammatory and immunomodulatory activities while reducing the intrinsic toxicity of diterpenoid components such as triptolide (Figure 4).

**Figure 5:**
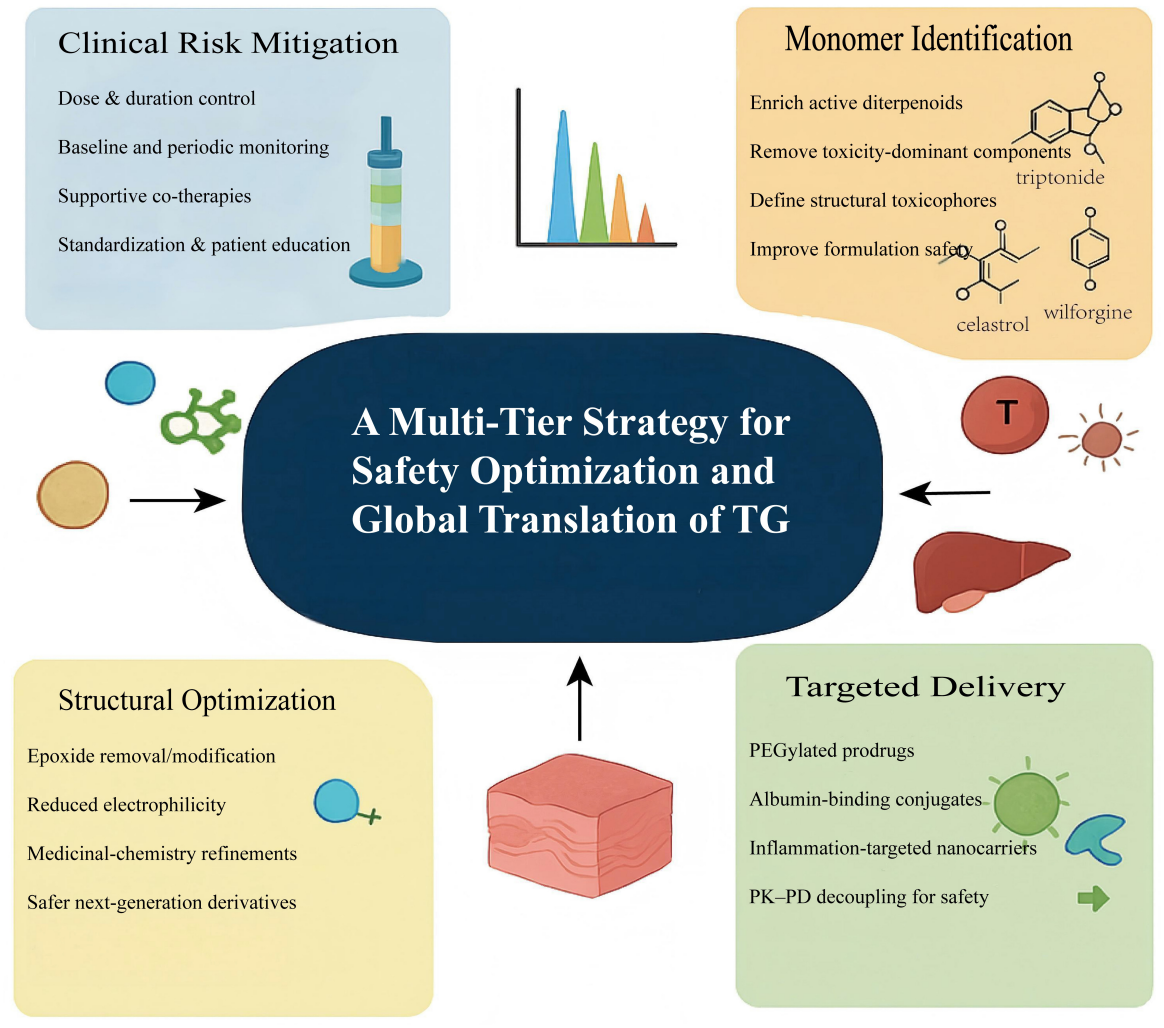
This figure summarizes the four interconnected pillars supporting the development of safer and more translatable TG-based therapeutics. Clinical risk mitigation focuses on dose control, structured monitoring, supportive co-therapies, and standardized patient education. Monomer identification differentiates efficacy-driving diterpenoids from toxicity-dominant components and defines key structural toxicophores to improve formulation-level safety. Structural optimization employs rational chemical modifications—such as epoxide removal, reduced electrophilicity, and targeted medicinal-chemistry refinements—to generate next-generation derivatives with lower systemic toxicity. Targeted delivery strategies, including PEGylated prodrugs, albumin-binding conjugates, and inflammation-responsive nanocarriers, help decouple pharmacodynamic activity from systemic exposure and broaden the therapeutic window. Together, these four dimensions outline a coherent roadmap for advancing TG derivatives toward safer clinical application and global translation.

First, monomer identification has become a foundational strategy for improving safety. Recent analytical advances—including bioactivity-guided fractionation, metabolomic profiling, and network-based target analysis—have demonstrated that only a small subset of TG diterpenoids meaningfully contributes to therapeutic activity (83), while many structurally reactive constituents are responsible for disproportionate toxicity. Studies consistently show that key bioactive monomers such as triptolide, celastrol, tripdiolide, and wilforgine possess defined immunomodulatory and anti-inflammatory mechanisms, whereas epoxide-rich or highly electrophilic diterpenoids exhibit stronger hepatotoxic (84), reproductive, and hematologic toxicity due to their high chemical reactivity and low target selectivity. Structural analyses further reveal that the C14–C15 epoxide of triptolide represents a major toxicophore, and its removal yields derivatives with markedly improved safety (67). By distinguishing efficacy-driving monomers from toxicity-dominant components, monomer identification enables selective enrichment of beneficial constituents and reduction of harmful ones, effectively widening the therapeutic window at the formulation level (Figure 5).

Second, structural optimization of key diterpenoids has become the most effective strategy to intrinsically suppress toxicity. SAR studies consistently identify the C14 α-epoxide of triptolide as the dominant toxicophore, driving mitochondrial injury, oxidative stress, apoptosis, and reproductive toxicity (68, 85). Removing or modifying this epoxide—as exemplified by triptonide—markedly reduces systemic toxicity while preserving core immunosuppressive activity. Selective hydroxylation represents another successful approach. LLDT-8 maintains strong inhibition of NF-κB and STAT3 but induces substantially less oxidative and mitochondrial stress, confirming that fine-tuning reactive functional groups can widen the safety margin without compromising efficacy (86). Beyond these representative derivatives, additional medicinal-chemistry refinements—such as lactone-ring modification, redox-group replacement, and stereochemical adjustments—have produced compounds with lower electrophilicity, improved metabolic stability, and reduced off-target reactivity (85). These structural strategies, together with emerging targeted-delivery and prodrug systems, have enabled several optimized derivatives to advance into preclinical or early clinical evaluation, underscoring their promise as safer next-generation Tripterygium agents (85, 86).

Third, multiple delivery platforms—including PEGylated triptolide prodrugs (87), albumin-binding conjugates, and inflammation-responsive nanoparticles—have been shown to markedly reduce peak systemic exposure while enhancing tissue selectivity (88). PEGylated and albumin-binding formulations extend circulation time and modulate release kinetics, thereby lowering hepatic accumulation and minimizing off-target toxicity (88). Inflammation-targeted nanocarriers further improve drug deposition in diseased tissues by exploiting microenvironmental cues such as enhanced vascular permeability and macrophage uptake (87). Together, these engineered systems effectively decouple pharmacodynamic activity from systemic exposure, enabling therapeutic concentrations to be achieved at inflamed or fibrotic sites with substantially reduced systemic toxicity and thereby broadening the therapeutic window of triptolide-based agents (89).

Together, these optimization strategies illustrate a clear developmental trajectory toward safer TG-derived therapeutics. By combining monomer-level refinement, rational structural redesign, and advanced delivery technologies, next-generation TG derivatives are progressively overcoming the intrinsic toxicity barriers that have historically limited their clinical adoption.
